# Anomaly Detection of Permanent Magnet Synchronous Motor Based on Improved DWT-CNN Multi-Current Fusion

**DOI:** 10.3390/s24082553

**Published:** 2024-04-16

**Authors:** Minqi Tang, Lihua Liang, Haitao Zheng, Junjun Chen, Dongdong Chen

**Affiliations:** 1 College of Mechanical Engineering, Zhejiang University of Technology, Hangzhou 310023, China; 2112102117@zjut.edu.cn (M.T.); zht_111963@163.com (H.Z.); 1112002007@zjut.edu.cn (J.C.); 2 Key Laboratory of Special Equipment Safety Testing Technology of Zhejiang Province, Zhejiang Academy of Special Equipment Science, Hangzhou 310020, China; chendd@zjtj.org

**Keywords:** DWT-CNN, anomaly detection, multi-current signals, permanent magnet synchronous motor

## Abstract

The Permanent Magnet Synchronous Motor (PMSM) is the power source maintaining the stable and efficient operation of various pieces of equipment; hence, its reliability is crucial to the safety of public equipment. Convolutional Neural Network (CNN) models face challenges in extracting features from PMSM current data. A new Discrete Wavelet Transform Convolutional Neural Networks (DW-CNN) feature with fusion weight updating Long Short-Term Memory (LSTM) anomaly detection is proposed in this paper. This approach combines Discrete Wavelet Transform (DWT) with high and low-frequency separation processing and LSTM. The anomaly detection method adopts DWT and CNN by separating high and low-frequency processing. Moreover, this method combines the hybrid attention mechanism to extract the multi-current signal features and detects anomalies based on weight updating the LSTM network. Experiments on the motor bearing real fault dataset and the PMSM stator fault dataset prove the method’s strong capability in fusing current features and detecting anomalies.

## 1. Introduction

Permanent Magnet Synchronous Motors (PMSM) are widely used as a power source in all kinds of machinery and equipment, and, once an abnormality occurs, the equipment stops running if it is light, or the workers who operate the equipment are injured if it is heavy, so the detection of abnormalities in PMSM is crucial. Existing permanent magnet synchronous motor fault diagnosis research focuses on the lack of permanent magnet synchronous motor such as elevator industry research [1,2]. This paper aims to study the multi-current fusion of permanent magnet synchronous motor fault diagnosis method. Faults in permanent magnet synchronous motors are generally categorized into four types: (1) bearing faults, (2) stator faults, (3) rotor faults, and (4) magnetic path faults [3]. Intelligent anomaly detection for permanent magnet synchronous motors using deep learning has achieved remarkable results due to advances in sensor technology and data-driven techniques [4].

Currently, mainstream methods for detecting anomalies in permanent magnet synchronous motors employ signals such as raw vibration [5], current [6], magnetic field [7], and other signals. As vibration models can effectively reflect internal faults, Xu et al. [8] proposed an improved global contextual residual neural network for motor anomaly detection by extracting vibration signal features. Zeng et al. [9] analyzed the spatiotemporal distribution characteristics of the stator magnetic flux to extract rotor features, enabling online detection of the motor rotor faults. Mbo’o et al. [10] utilized current characteristics generated by the stator current spectra for bearing fault diagnosis.

Different sensor signals have various advantages in fault diagnosis. However, in practical applications, non-invasive current signals have the advantages of easy acquisition, high accuracy, and minimal sensitivity to other components due to the noise interference of vibration signals from the environment, such as noisy machine rooms and magnetic sensitivity to changes in magnetic fields. Researchers more frequently investigate such methods.

Zhang et al. [11] employed the hypergraph neural network to mine the direct correlation of multiple nodes of current signals. Moreover, the authors also employed hyperedge convolution to capture high-order feature relationships. Zhuo et al. [12] used the multi-sensor information-driven attention mechanism and the improved Adaboost method to train different signals, evaluating signal sensitivity to enhance diagnostic efficiency. However, most CNN-based methods fail to explore hierarchical representations from measured current signals. Such methods solely utilize features from the final convolutional layer for fault recognition, leaving some useful hierarchical features extracted from the intermediate layers unexplored and underutilized. Furthermore, these methods lack a fault signal learning mechanism, i.e., there is no model updating and indiscriminate processing of signals (including all pulses and patterns) in a single pass, making them prone to signal learning overfitting. Since different features have a different importance for the fault detection task, the network should focus more on extracting discriminative features.

To improve the fault feature extraction ability of CNN applied to the fault monitoring system of the appropriate amount of computation—so that it has an explicit physical meaning and mechanistic significance, as well as relationships between preceding and succeeding signals—scholars have introduced traditional signal analysis methods, Recurrent Neural Networks (RNN) methods [13], and federal learning methods into the CNN. Li et al. [14] designed a continuous wavelet convolution layer to replace the first convolution layer of the standard CNN. Hence, the CNN gradually incorporates the advantages of the wavelet transform to extract additional fault features in bearing failures. Chen et al. [15] replaced the fully connected CNN layer with LSTM when investigating grid anomalies. Moreover, the authors used the gating mechanism and timing characteristics of LSTM to extract additional internal fault features. Gao et al. [16] proposed federated learning combined with reinforcement learning to train the measured signals in a distributed manner, which not only reduces the amount of model computation but also guarantees data security. The solutions mentioned above have achieved favorable outcomes in their respective fields. However, as low-speed, high-torque permanent magnet synchronous motors for industrial use require precise position control, the resulting faults are slightly different from those of ordinary permanent magnet synchronous motors, which are more prone to asymmetrical three-phase currents due to noise and other causes [17]. In practical engineering, the cost-saving of detecting the power of only two out of the three phases will be used when detecting the motor currents, deducing information about the third phase [18]. Although K Jankowska et al. [19] proposed fault diagnosis methods for two-phase currents, calculating the residual differentials of currents and currents in the DC bus, the residual differentials contain less information about other faults. Hence, the above solutions do not take into account, for example, low-speed, high-torque permanent magnet synchronous motor application scenarios.

A new DW-CNN feature fusion-weighted updating LSTM classification network is proposed in this paper to address the issue of CNN networks struggling to effectively extract current features from PMSMs and achieve abnormal detection under non-steady-state conditions. The proposed method integrates cyclical wavelet packets and CNN to extract high and low-frequency features of two-phase or three-phase current signals. The weighted updating LSTM network enhances the accuracy of abnormal detection of PMSMs under various operating conditions and eliminates interference caused by incomplete symmetry in three-phase currents. An overview of the main work in this paper is as follows.

A DW-CNN feature fusion method with separate processing of high and low frequencies is proposed to fuse different current signals and perform feature processing for targeted high and low frequencies. The high- and low-frequency signal features of the current signals are obtained based on wavelet packet transform. Moreover, the noise-resistant and strong multiscale features are obtained by simple coding-based CNN and direct CNN, laying the foundation for the subsequent model.

The circular attention mechanism is incorporated into the network to guide the model and target the inconspicuous frequency and time features of the multi-current signal. The multi-current signal is transformed via a wavelet-convolution-attention loop to form a complete fusion feature signal rich in time-domain frequency.

A weighted updating LSTM anomaly detection method is introduced in this paper based on the improvements above. The model is referred to as the Multilayer Wavelet Attention LSTM Convolutional Neural Network (MWAL-CNN) model. The proposed method is verified on the induction motor test beds and industrial motor pumping system datasets. The experimental results demonstrate that, compared to five state-of-the-art methods, the proposed model achieves higher diagnostic accuracy under non-steady-state conditions, affirming the effectiveness of the proposed approach.

The remainder of the paper is organized as follows. The relevant theoretical background is briefly reviewed in Section 2. The details of the proposed method are explained in Section 3. The effectiveness of the proposed MWAL-CNN is validated in Section 4 against two experimental datasets with two-phase and three-phase current signals. Lastly, a discussion on the feasibility of the method for anomaly detection of permanent magnet synchronous motors is provided in Section 5.

## 2. Theoretical Background of Common CNN Modules

### 2.1. Standard CNN Modules

CNNs specifically designed for processing grid-structured data have gained widespread recognition in numerous computer vision and signal-processing applications [20]. The primary architecture of a CNN comprises an input layer, convolutional layers, pooling layers, fully connected layers, and an output layer. The convolutional layer is the core component of a convolutional neural network. Convolutional operations can be employed to extract local features from data. Each convolutional kernel has a small receptive field. However, when multiple convolutional layers are stacked together, the receptive field of the entire network is much larger. A CNN without fully connected layers is a simple linear transformation similar to a multilayer perceptron, limiting the network’s ability to handle complex data. Therefore, activation functions incorporating nonlinear transformation capabilities into the network are usually performed simultaneously with convolution operations. Commonly used activation functions include Sigmoid, Tanh, ReLU, and Leaky ReLU functions. The pooling layer is a nonlinear down-sampling operation of the features extracted from the convolutional layer. Commonly used pooling layers include maximum and average pooling layers. The implementation of the pooling layer can reduce the parameters, mitigating overfitting, and reducing the computational cost of the network.

### 2.2. Discrete Wavelet Transformation DWT

Discrete Wavelet Transformation (DWT) [21] was introduced to overcome the limitations of the Fast Fourier Transform (FFT) [22] which only allows for the extraction of the frequency components of the signal, eliminating information about the temporal localization of the frequency components, and also to overcome the limitations of the optimal window size selection for the Short Time Fourier Transform (STFT). It allows for time localization of the frequency components occurring in the signal and for the extraction of their time evolution. This feature makes it possible to detect characteristic patterns in the evolution of these components [23].

The DWT is transformed from the Continuous Wavelet Transformation (CWT). The formula for the CWT of the time function *f*(*t*) is:

(1) $$T(a,b)=\int_{-\infty }^{\infty }f(t)\frac{1}{\sqrt{a}}\psi (\frac{t-b}{a})(a,b\in R,a\neq 0)$$

$$\begin{array}{l} T(a,b):the\;transformed\;signal\\ a,b:adjust\;the\;frequency\;and\;the\;time \end{array}$$

DWT is obtained by replacing *a* and *b* with discrete points $2^{j}$ and $2^{j}k$, respectively, and the replacement part is given by:

(2) $$\psi_{\left(j,k\right)}(t)=\frac{1}{\sqrt{2^{j}}}\psi (\frac{t-2^{j}k}{2^{j}})$$

The above equation is also expressed as:

(3) $$f(t)=\sum_{j=-\infty }^{\infty }\sum_{k=-\infty }^{\infty }C_{j,k}\psi_{\left(j,k\right)}(t)$$

(4) $$C_{j,k}=\int_{-\infty }^{\infty }f(t)\psi_{\left(j,k\right)}^{*}(t)dt$$

We called $C_{j,k}$ the wavelet coefficients, $\psi_{\left(j,k\right)}^{*}$ and $\psi_{\left(j,k\right)}$ are conjugate relations.

Finally, the original time function can be written as:

(5) $$d_{j,k}=\int_{-\infty }^{\infty }f(t)\frac{1}{\sqrt{2^{j}}}\psi^{*}(\frac{t-2^{j}k}{2^{j}})dt$$

$$d_{j,k}:scaling\;coefficients\;$$

(6) $$d_{j,k}=\sum_{i=-\infty }^{\infty }h(k-2i)d_{j+1,i}+\sum_{i=-\infty }^{\infty }g(k-2i)C_{j+1,i}$$

$$\begin{array}{l} h,\;g:\;low-pass\;and\;high-pass\;filters\;derivedfrom\;the\\ \;original\;analyzing\;wavelet \end{array}$$

**Figure 1:**
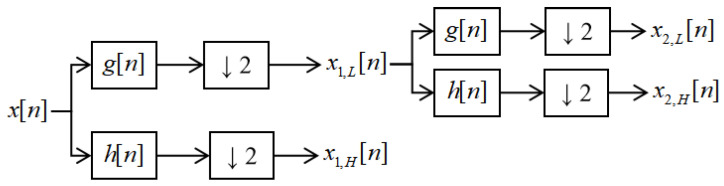
The decomposition structure of wavelet packet analysis.

When the real sampled signal *x*[*n*] (*x*1[*n*], *x*2[*n*], *x*3[*n*]...) is decomposed layer by layer after being input into the DWT, the j layer can be obtained from the above equation, as shown in Figure 1:

(7) $$x_{j,L}[n]=\sum_{k=0}^{k-1}x[2n-k]g[k]$$

(8) $$x_{j,H}[n]=\sum_{k=0}^{k-1}x[2n-k]h[k]$$

For a detailed description of the symbols in the diagram presented in Figure 1, see Table A2.

### 2.3. Hybrid Attention Mechanism

**Figure 2:**
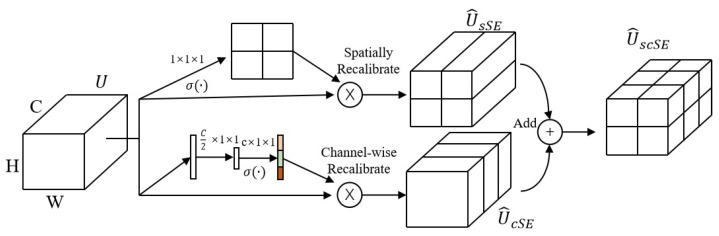
The architecture details of scSE.

The attention mechanism [24] in deep learning mimics the human visual and cognitive system by allowing the neural network to focus on the relevant parts of the input data as processed, as shown in Figure 2. The neural network is able to automatically learn and selectively focus on the important information in the input by introducing an attention mechanism, improving the model’s performance and generalization. The Hybrid Attention Mechanism (scSE module) is commonly used in image semantic segmentation. The mechanism comprises two parallel parts: Channel Attention (sSE module) and Spatial Attention (cSE module). The sSE module is located in the upper half, while the cSE module is in the lower half. This architecture enhances meaningful features, suppresses irrelevant features, and smoothens segmentation boundaries. For a detailed description of the symbols in this diagram, see Table A2.

### 2.4. Standard LSTM

**Figure 3:**
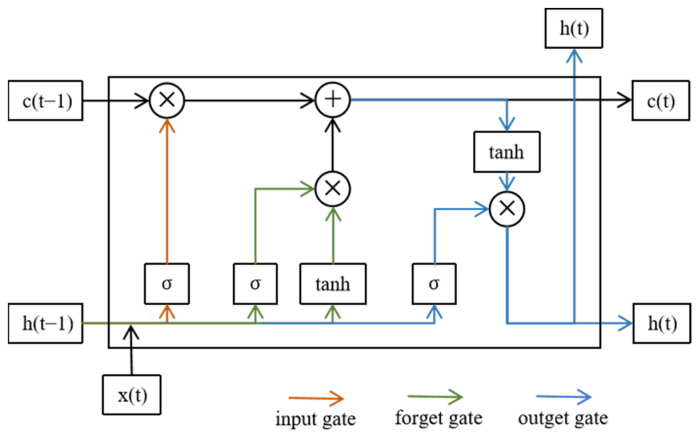
The architecture details of LSTM.

LSTM [25] was designed to address the issue of long-term dependencies commonly found in general Recurrent Neural Networks (RNN). Using LSTM effectively allows for the transmission and representation of information in long sequences without causing useful information from the distant past to be ignored. Simultaneously, the LSTM can also solve the gradient vanishing and gradient explosion problems in RNN. The LSTM has now found widespread application in anomaly detection. As shown in Figure 3, LSTM comprises three intelligent units: the input gate (the green line), the forget gate (the orange line), and the output gate (the blue line). For a detailed description of the symbols in this diagram, see Table A2.

The input gate inputs from the current layer and the previous layer’s hidden state computes which inputs to remember and stores them. The forget gate determines which information to forget or discard, depending on the current hidden state and the current input. Finally, the output gate decides which information to output, depending on the input to the current layer’s neurons and the previous layer’s output.

## 3. LSTM Anomaly Detection with DW-CNN Weight Updating

A detailed introduction to the DW-CNN feature fusion and weight-updated LSTM classification network used for motor anomaly detection under non-stationary conditions is provided in this section.

### 3.1. MWAL-CNN Structure

**Figure 4:**
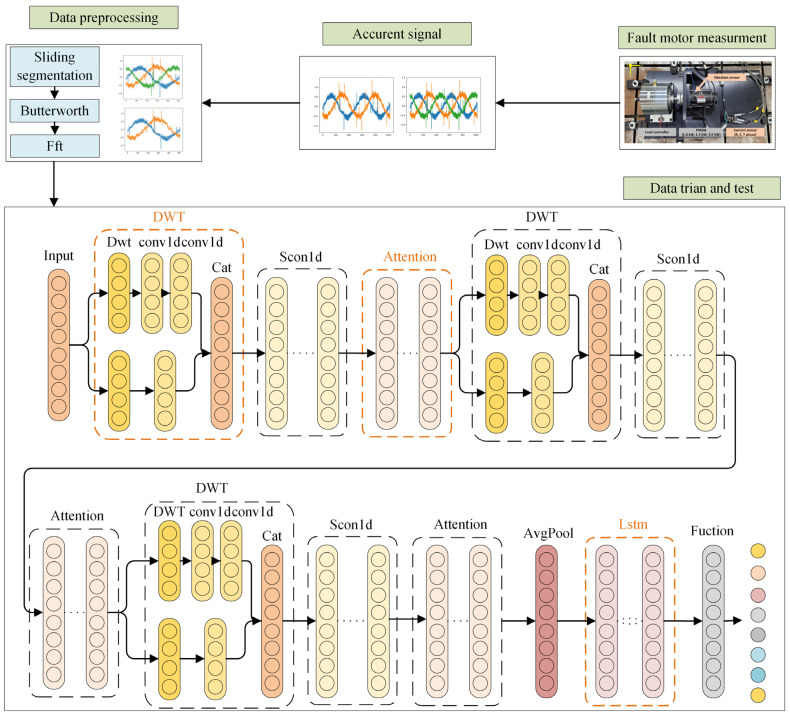
Framework of the proposed method.

The MWAL-CNN structure is shown in Figure 4 and it comprises four parts, i.e., the high- and low-frequency DWT layer, the standard CNN layer, the hybrid attention mechanism layer, and the weight update LSTM layer. For a detailed description of the symbols in this diagram, see Table A1 and Table A2. The first three parts are combined into one and looped three times to connect with the weight-updating LSTM layer to form a looped DW-CNN structure. The DWT and CNN layers remove the high-frequency noise component in the multi-current signal and amplify the low-frequency component containing many features. The hybrid attention mechanism layer extracts the inconspicuous frequency signals and time signals in the high- and low-frequency signals. Lastly, the LSTM layer of weight updating classifies the anomalous faults from the extracted feature signals. The specific steps are as follows.

Step 1: Data preparation. The three-phase current signals of the induction motor are collected in different operating states. The long signal data from each sensor is divided into windows to enhance the number of samples. Each window contains at least one period of sinusoidal signals. Each divided signal is preprocessed with Fast Fourier Transform and Butterworth Filter and divided into training and test samples. The Fourier transform can extract the frequency domain features of the current information and other filtering methods, such as the Butterworth filter and the Chebyshev filter, that have the advantage of maximum smoothing of the frequency response curve in the passband.

Step 2: Training process. The initial weights of the LSTM network are determined, the preprocessed data are input into the MAWL-CNN model for training, and the fusion features after three layers of the network’s feature extraction are obtained. The fused features are mapped to different fault categories via LSTM, and LSTM weights are updated until optimal weights are obtained.

Step 3: Validation procedure. Test samples are placed into the trained model to test its reliability and the response degree, and evaluation metrics (such as accuracy) are used to evaluate the model.

More details are discussed in Section 3.2, Section 3.3 and Section 3.4.

### 3.2. DWT Structure for Simple Coding

**Figure 5:**
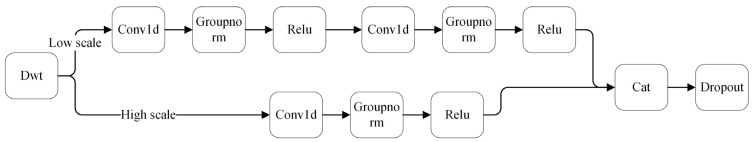
The architecture details of the proposed DWT layer.

The combination of one-dimensional Discrete Wavelet Transform and CNN is characterized by excellent signal feature extraction capabilities. It can decompose the signal into low- and high-frequency components. Additional processing of these two components can be used for denoising and amplifying hidden information. The commonly used DWT method combined with CNN is as follows. Through the DWT layer decomposition of the signal into high- and low-frequency components, where the components or processed components are treated through the cat function and turned into the original signal dimensions consistent with the DWT layer or CWT layer instead of the first convolutional layer, this method of extracting the features of the ability to be limited cannot be processed for the subsequent features. For this reason, this paper proposes a new DWT layer to be used to extract the high- and low-frequency components, respectively, through the combination of the DWT layer and the CNN layer into a single unit to replace each convolutional layer [26]. As shown in Figure 5 and Table A2, when combined with the multiscale attention mechanism described in Section 3.3, the new DWT layer learns information hidden in the time and frequency domains of multi-current signals.

Once the DWT layer decomposes the signal into high- and low-frequency components, the high-frequency component reduces the noise through the one-dimensional convolution of a larger kernel. Moreover, the low-frequency component is formed through the one-dimensional convolution of two smaller kernels to form a simple coding and decoding and a low-frequency feature map with clearer features. A dropout layer is added to enhance robustness after recombining the high- and low-frequency components. The feature models of the low-frequency components can be extracted by different processing of high- and low-frequency components decomposed by DWT to reduce the high-frequency noise generated by the PMSM.

### 3.3. Hybrid Attention Mechanisms

**Figure 6:**
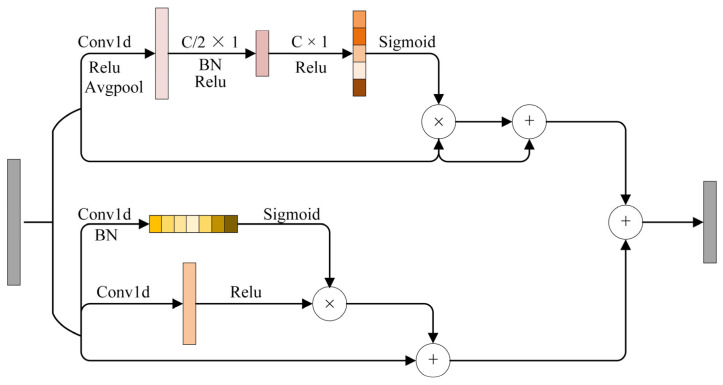
The architecture details of the proposed hybrid attention mechanism layer.

The hybrid attention mechanism is effective in the visual domain, and it plays a similar role in fault diagnosis. The structure of the hybrid mechanism layer (Figure 6 and Table A2) comprises two attention layers, i.e., the Channel Frequency Attention Mechanism (FAM) layer and the Time Attention Layer. The FAM layer effect is consistent with that of the channel attention layer in the visual domain. The core idea of FAM is to operate in conjunction with the self-learning ability of CNN to filter out the most useful channel frequency features from the mixed features. The FAM layer first inputs the feature into a 1 × 1 convolution to reduce the input feature aliasing effect. Then, the FAM layer introduces avgpool to compress the global information into a channel representation vector. Lastly, the FAM layer maps the cross-channel feature information through two 1 × 1 convolution operations. The layer introduces a sigmoid function to map the feature vector into the 0–1 interval to form a feature weight vector. Finally, the FAM layer introduces a residual block to optimize the backward propagation.

The time attention layer effect is consistent with that of the spatial attention layer. The core idea is to leverage useful temporal features extracted by different CNNs. The time attention layer consists of two 1 × 1 convolutions to obtain different weight values, introducing ReLU and normalization functions. Finally, the input and the weighted features are combined using a matrix multiplication to obtain the final time feature.

The combination of the results from both FAM and time attention layer forms a hybrid attention mechanism. FAM extracts the frequency features of the current signal in detail, especially inconspicuous features. On the other hand, the time attention layer focuses on the temporal features of the current signal, guiding the feature learning of the CNN model to complete channel and time aspects and ensuring comprehensive feature extraction.

### 3.4. LSTM with Weight Update

**Figure 7:**
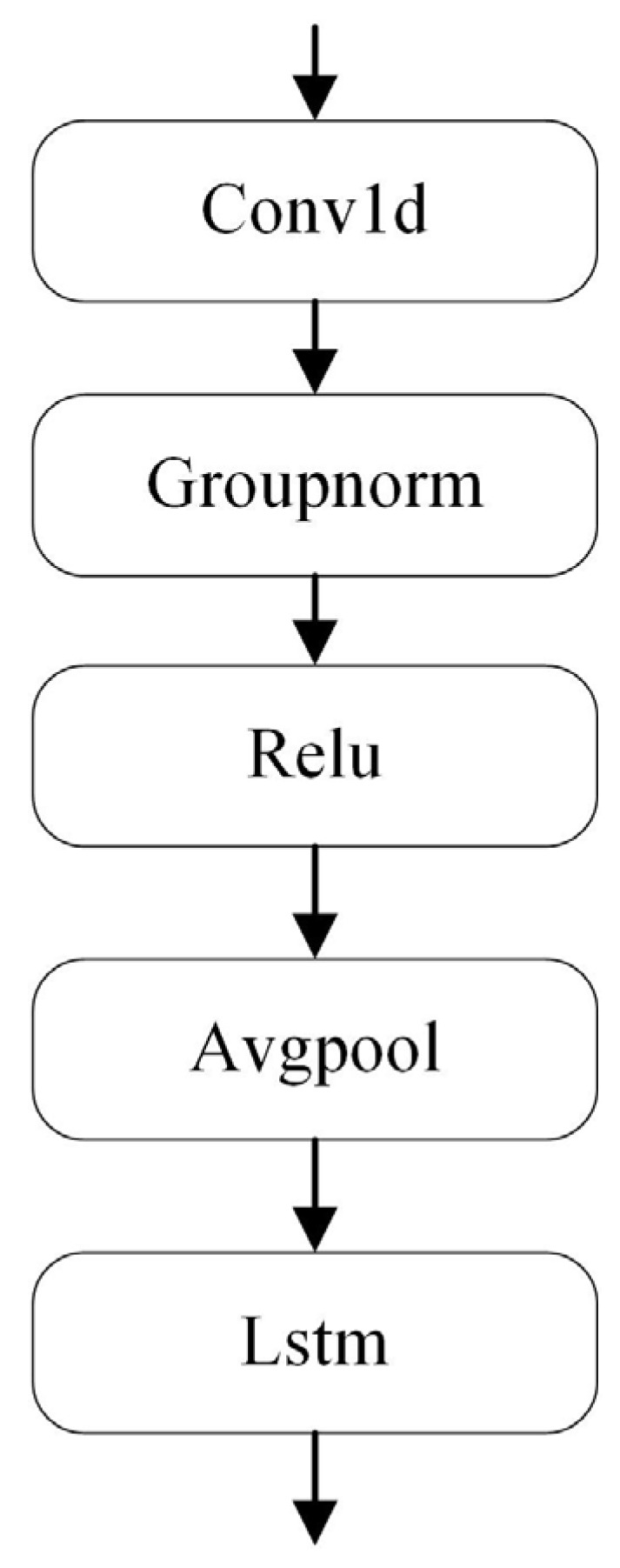
The architecture details of the proposed LSTM layer.

The classifier for anomaly detection comprises a one-dimensional convolutional layer, a weight-updated LSTM layer, and full connectivity to fuse the linear mapping between features and outputs, as shown in Figure 7 and Table A2. Synchronous motor anomalies of a permanent magnet tend to arise gradually. Moreover, there is a great correlation before and after anomaly occurrence. Lastly, the hidden layer of the LSTM can connect these correlated features and select the optimal hyperparameters.

## 4. Method Validation

### 4.1. Experimental Setup and Evaluation Metrics

The proposed MAWL-CNN was tested on a computer with Intel i5-12600kf CPU and NVIDIA GTX 3060ti GPU equipped with pytorch library and pytorch wavelet [27]. Several performance metrics [28] such as accuracy, precision, recall, and F1 score were used to evaluate the proposed method. Accuracy is the ratio of correctly predicted samples to the total samples. At the same time, precision refers to the ratio of the proportion of actually positive samples among those predicted to be positive to the proportion of samples that are predicted to be positive. Recall refers to the proportion of samples that are actually positive among those that are predicted to be positive, and the F1 score denotes the classification accuracy of each category in the anomaly detection task. In addition, the single epoch training time was chosen as a computational complexity evaluation metric to evaluate the computational burden of the model. Four state-of-the-art methods and a single machine learning method were chosen as the basis for comparison with MWAL-CNN: a Multilayer Wavelet Attention Convolutional Neural Network (MWA-CNN) [29], a Multiattention One-Dimensional Convolutional Neural Network (MA1DCNN) [30], a One-Dimensional Convolutional Neural Network (1DCNN) [31], a Deep Residual Network (ResNet18), and a Multilayer Perceptron (MLP). The training strategy for these five methods is the same as that proposed for the MWAL-CNN. Each method was executed five times in the experiment to reduce the randomness of the results and to verify the model’s robustness.

Two datasets, i.e., the real damaged motor bearing dataset and the vibration and current dataset of a three-phase permanent magnet synchronous motor with stator faults, were used in the experiments to evaluate the performance of the proposed method. The motor bearing data were obtained from the University of Paderborn, Germany, and the vibration and current dataset of a three-phase permanent magnet synchronous motor with stator faults were obtained from the Korean Academy of Sciences. Cross entropy loss and Adam’s algorithm were used in the training process, where the motor bearing data learning rate and batch size were set to 0.008 and 150, respectively. The data learning rate and batch size were set to 0.01 and 150, respectively, due to the more distinctive characteristics of the applicable current faults. Moreover, the data were not preprocessed. The mother wavelet used in this paper was the Daubechies (db) wavelet. Other wavelets, such as Haar and Dmeyer, were tested. However, their effect was slightly lower than that of the db wavelet.

### 4.2. Anomaly Detection of the Real Bearing Fault Data Set

#### 4.2.1. Data Description

**Figure 8:**
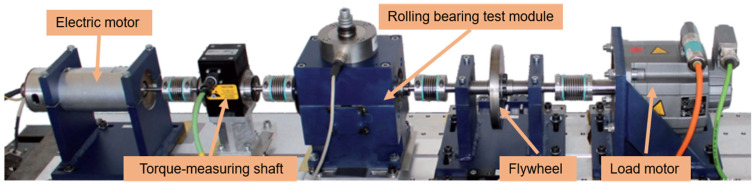
Motor Fault Diagnosis Platform [30].

As previously mentioned, the experimental data were obtained from the acquisition test bed for the motor bearing signal at the University of Paderborn (PU), Germany [32]. The test stand comprised several modules (shown in Figure 8 in order from left to right): an electric motor, a torque-measuring shaft, a rolling bearing test module, a flywheel, and a load motor. The test rig generated experimental data by mounting ball bearings characterized by various damage degrees in the bearing test module. The faulty bearings were classified into artificial damage and real damage. Only the real damage bearing data were used in this paper to better test the practicality of the model in real scenarios. The real damage was obtained via an accelerated life test rig, which comprised a bearing housing and an electric motor, providing power to the shafts of four type 6203 test bearings in the housing. The test bearings rotated under the radial load applied by a spring-screw mechanism. Failures included three types.

Single-point damage (single damage): a single component of a rolling bearing is affected by a single damage, such as a pitting pit on the inner ring.

Repetitive damage: the same damage symptoms in the same bearing parts of several places repeatedly; for example, the inner ring has more than one non-continuous pitting.

Multiple damage: different ring-breaking symptoms occur in different parts of the same bearing, such as pitting pits on the inner and outer rings.

These three types of damage comprise a total of 14 categories. Detailed parameters are shown in Table 1.

The sampling frequency of the current and vibration signals used in this experiment was 64 kHz, and the sampling frequency of mechanical parameters (loading force, loading torque, speed) was 4 kHz. Specifically, the dataset chosen for the analysis was “N09_M07_F10,” comprising 20 samples for each fault type, with each sample having a length of 256,823 data points. The sliding window function increased the signal samples. The length of each sample was 2048 × 1, and the training and test samples were divided according to the ratio of 7:2:1. Thus, a total of 24,544 training samples, 7012 validation samples, and 3508 test samples were obtained.

#### 4.2.2. Comparative Analysis

Experiments were conducted on MWAL-CNN, four state-of-the-art models, and a single machine-learning model, with the results shown in Table 2. The network using convolutional networks as the main body outperformed the machine learning representative MLP in every way. Of the five convolutional main body models, the proposed MWAL-CNN exhibited a high accuracy of 99.772% and an F1 score of 99.786%. From the time, it can be seen that the proposed model exhibited a time difference of 1 s relative to the other advanced models, and 1DCNN and MLP required less time due to the simplicity of their models, thus achieving satisfactory results in the fault diagnosis task.

**Figure 9:**
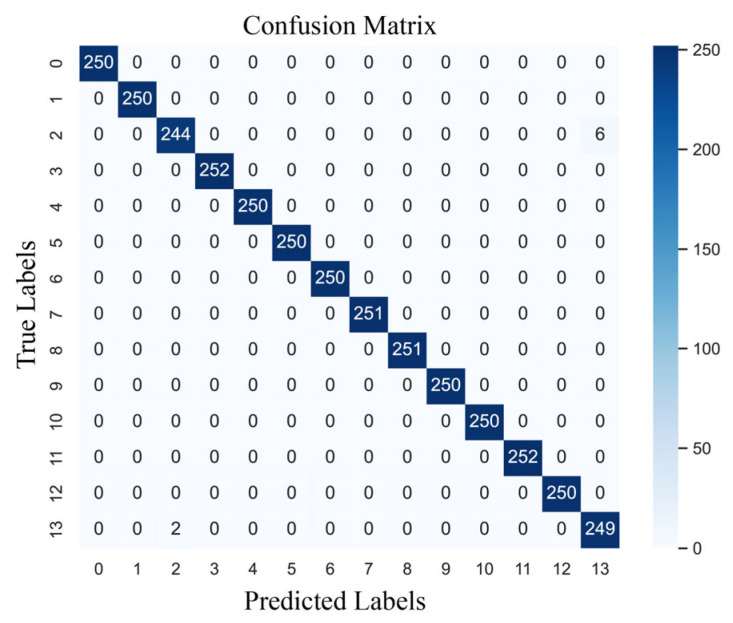
The confusion matrix of MWAL-CNN on the PU dataset.

The confusion matrix for one of the training models in this paper is shown in Figure 9, with the values along the diagonal indicating the number of correct samples for each category. There was only a single classification error in category 12, while the rest were correctly classified. Therefore, it can be concluded that the model obtains good detection results for most of the real bearing faults of motors.

**Figure 10:**
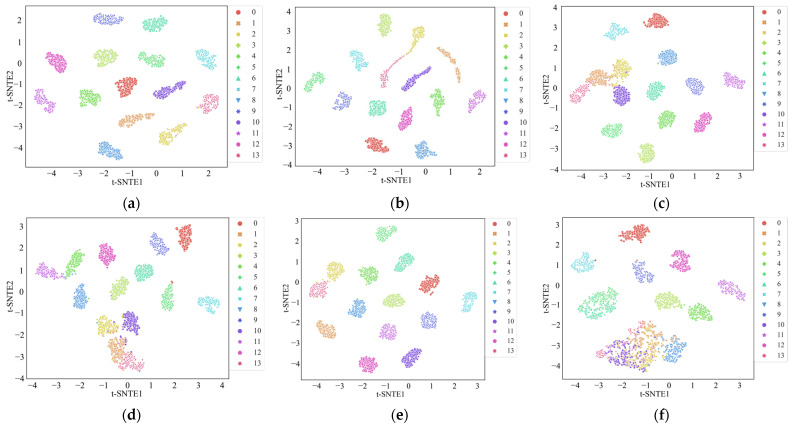
The visualization of the features of the fully connected layer using t-SNE on the PU dataset. (**a**) MWAL-CNN, (**b**) MWA-CNN, (**c**) MA1DCNN (**d**) 1DCNN, (**e**) ResNet, (**f**) MLP.

The t-distributed Stochastic Neighbor Embedding (t-SNE) algorithm was introduced to visualize the distribution of the results from the six methods and provide more intuitive results [33]. In a good classification model, the high-dimensional features extracted in the intermediate layers should separate features into a lower dimension, allowing a visual assessment of feature clustering. As shown in Figure 10, each color represents a different state of the motor. It is evident that, compared to the other five methods, the feature distribution map associated with the MWAL-CNN almost entirely distinguishes between each category. Furthermore, the other networks exhibited some instances of overlapping categories or misclassification. This observation indicates that the MWAL-CNN model has better discriminative power, confirming the superiority of the proposed method.

#### 4.2.3. Effectiveness of Multiple Current Signals

**Figure 11:**
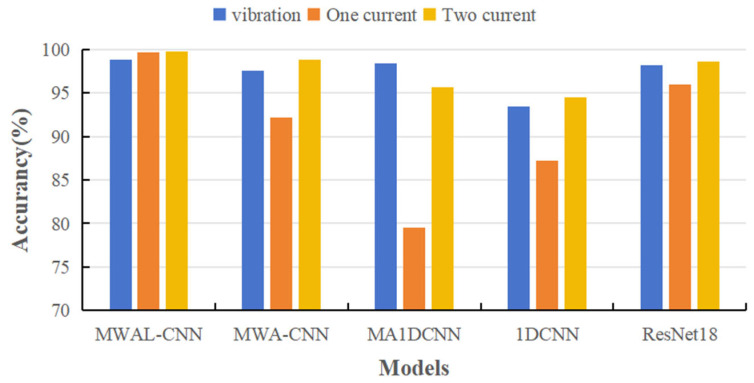
The experimental results from the five methods for different signals on the PU dataset.

Four types of advanced models and the proposed model (a total of five model types) were selected in this paper to verify the effectiveness of multi-current fusion. A single vibration signal and multiple current signals were tested and compared across five model types. Each type of experiment was executed five times; only the current dimension was changed, while the remaining conditions remained the same. Only the accuracy rate and F1 score were used as evaluation indices. The results are shown in Table 3 and Figure 11.

It can be observed that most models worked well for a single vibration signal. Moreover, some models used a single current signal to correctly identify the true bearing abnormality of the motor with low accuracy. In addition, most models attained a high accuracy rate and F1 score for multi-current abnormality detection. It can be inferred that a single-current signal contains significantly fewer features than a vibration signal. Therefore, the anomaly detection of a single-vibration signal is stronger than that of a single-current signal. Moreover, fusing the features of a multi-current signal results in more features than the single-vibration signal, a phenomenon which can be interpreted as the validity of the multi-current signal.

#### 4.2.4. Validity of the Included Modules

Ablation experiments with six control variables were designed to verify the validity of the different modules used in the proposed model. All model structures were the same as those of the MWAL-CNN (except for the missing modules) and can be defined as follows:

(1) The original model with only a three-layer CNN has a structure containing only three one-dimensional convolutional modules and a DWT structure without high- and low-frequency processing. The model’s name is CNN.
(2) The three-layer CNN model is based on the DWT module containing high- and low-frequency processing. The model’s name is CNN-W.
(3) The three-layer CNN model is based on the hybrid attention mechanism module, denoted as CNN-A.
(4) The three-layer CNN model is based on the addition of a hybrid attention mechanism and a weight update for two modules of the LSTM and has no high- and low-frequency processing DWT structure, and the model is denoted as CNN-AL.
(5) The three-layer CNN model is based on adding two modules of DWT for high- and low-frequency separation and LSTM for weight update without a hybrid attention structure; the model’s name is CNN-WL.
(6) The three-layer CNN model is based on the addition of high-frequency separated DWT and two hybrid attention t-modules. No weight update is included for the LSTM structure, and the model is denoted as CNN-AW.

Since the combination of a three-layer CNN model and a hybrid attention mechanism module with few layers can easily cause overfitting, the case is not discussed in this paper. The experiments used the accuracy rate and F1 score as evaluation indices. The results are shown in Table 4, from which it can be evinced that the model CNN-W, with the addition of high- and low-frequency fused DWT structures, was able to improve the accuracy rate by 33.637% compared to a single three-layer CNN model. This percentage is significantly higher than that of the other two modules. Furthermore, the accuracy rate improvement of the added two modules containing high- and low-frequency fused DWT structures is greater than that of the other models, indicating that the high- and low-frequency fused DWT can fuse the current features. Moreover, its feature extraction ability is even greater than the sum of the hybrid attention mechanism and the weight update for LSTM. Adding only the hybrid attention module will cause the model to extract too many small features, resulting in overfitting.

Combining the DWT module with separate high- and low-frequency processing can force the model to play an important role in the extraction of inconspicuous features. For example, the accuracy of the CNN-AW model was 0.856% higher than that of other models, indicating that the high- and low-frequency fused DWT can fuse the current features. Moreover, its feature extraction ability was found to be even greater than the sum of the hybrid attention mechanism and the weight update for LSTM.

Adding only the hybrid attention module will make the model extract too many small features, resulting in overfitting. Combining the DWT module with separate high- and low-frequency processing can force the model to play an important role in the extraction of inconspicuous features. For example, the accuracy of the CNN-AW model was 0.856% higher than that of CNN-W, while the accuracy of the MWAL-CNN model was 1.824% higher than that of CNN-WL. Adding the LSTM module with weight updating improved CNN-L by 16.22% compared to CNN. On the other hand, the MWAL-CNN model improved CNN-AW by 1.482%, indicating that the addition of the LSTM module with weight updating further improves the anomaly classification ability of the CNN model.

**Figure 12:**
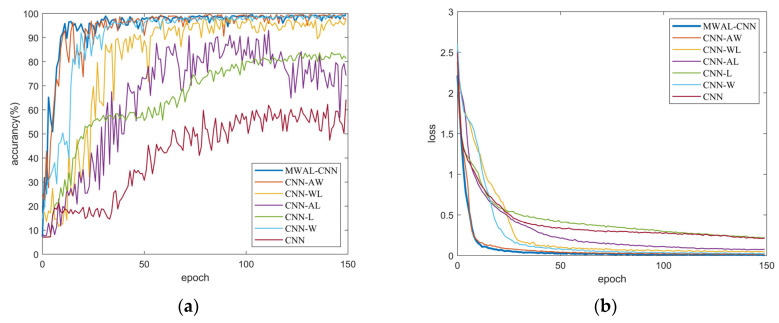
The validation accuracy curves and training loss curves of seven models on the PU dataset: (**a**) validation accuracy curve; (**b**) training loss.

The training loss and test accuracy curves of different models are provided in Figure 12. It can be seen that the highest accuracy was achieved when all three modules were added. In contrast, overfitting occurred when only the attention mechanism and weight update for the LSTM modules were added. Moreover, the oscillation amplitude of the curves with the inclusion of the high- and low-frequency frequency-separated DWT module is slightly larger than that of the other curves. The smaller the oscillation, the more stable the model.

According to the training loss curve, the convergence performance of the final model is close to that of the three-layer CNN model. In addition, the DWT model with high- and low-frequency separation converges faster than the others, indicating that the mixed attention mechanism module and the weight-updating LSTM module can improve the model stability. Lastly, the high- and low-frequency separation of the DWT module can improve the model convergence.

In summary, it can be concluded that the DWT layer can extract the obvious and non-obvious features of the current signal. The multiscale attention mechanism layer can fuse the features of multiple current signals, and the weight-updating LSTM layer can classify anomalous signals. Consequently, the CNN-LSTM model can obtain more favorable results.

### 4.3. Experiments on Stator Fault Data Set for Permanent Magnet Synchronous Motors

#### 4.3.1. Data Description

**Figure 13:**
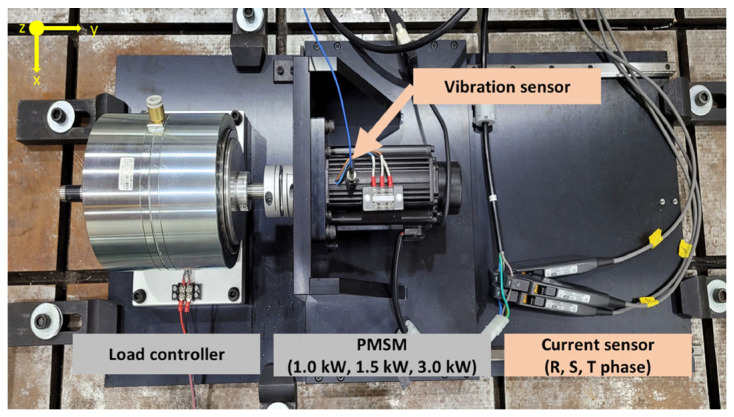
Description of the PMSM testbed [32].

The vibration and current datasets of a three-phase permanent magnet synchronous motor with stator faults collected by the Korea Institute of Science and Technology (KIST) were used for the experiments [34]. As shown in Figure 13, the test rig comprised a load controller made of hysteresis brakes, permanent magnet synchronous motor, and sensors. A hysteresis brake (AHB-10A) made by Valid Magnetics Ltd. can apply a torque load of up to 10 Nm to the PMSM. Flexible couplings and linear guides were used to prevent connection misalignment failures between the load controller and the PMSM. Experiments were conducted using three three-phase, four-pole permanent magnet synchronous motors from the same manufacturer with the same speed (3000 RPM), allowing for 5% of the torque limit of 15.1 Nm with three motors of different powers (1.0 kW, 1.5 kW, and 3.0 kW).

Each motor comprised 16 stator faults: eight inter-coil circuit faults and eight inter-turn circuit faults. Three motors with different kinds of faults were selected in this paper, each with two classes, i.e., a total of 12 classes of faults, as shown in Table 5. A total of 8593 training samples, 2455 validation samples, and 2456 test samples were obtained based on the 6:2:2 division of the training and the test sets and according to the window division of the increase in the data samples.

#### 4.3.2. Comparative Analysis

Similar to data 1, in this section, experiments were conducted on the stator fault dataset with MWAL-CNN and five state-of-the-art models, whose method configurations were explained in Section 4.1, to demonstrate the performance of these methods on different datasets. The anomaly detection results are shown in Table 6. It can be seen that this dataset has simpler fault types than the PU dataset and more one-phase current signals, obtaining a higher accuracy.

The proposed MWAL-CNN still obtained the highest recognition accuracy in each trial compared to the other five methods; calculation time increased by 0.4 s compared to the longest time. The minimum accuracy of MWAL-CNN was as high as 99.959% across five trials, providing satisfactory results in fault diagnosis tasks.

**Figure 14:**
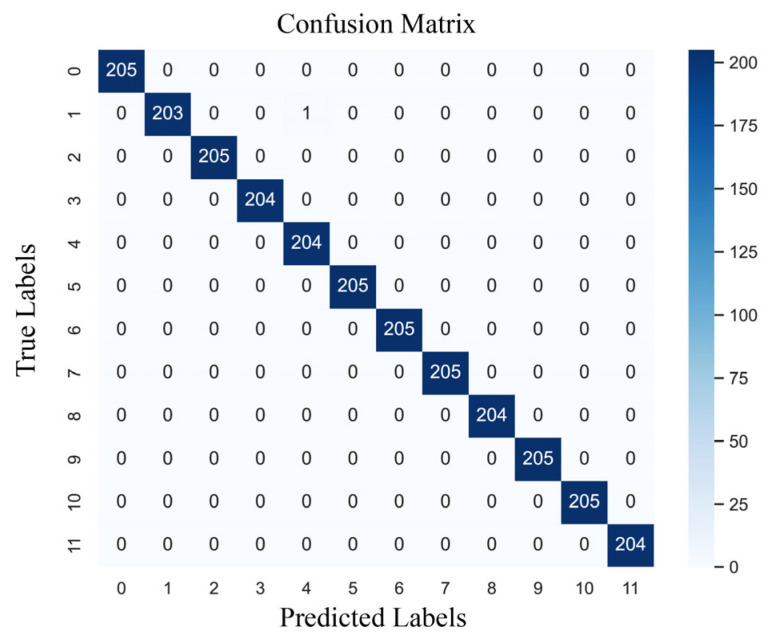
The confusion matrix of MWAL-CNN on the PMSM stator dataset.

**Figure 15:**
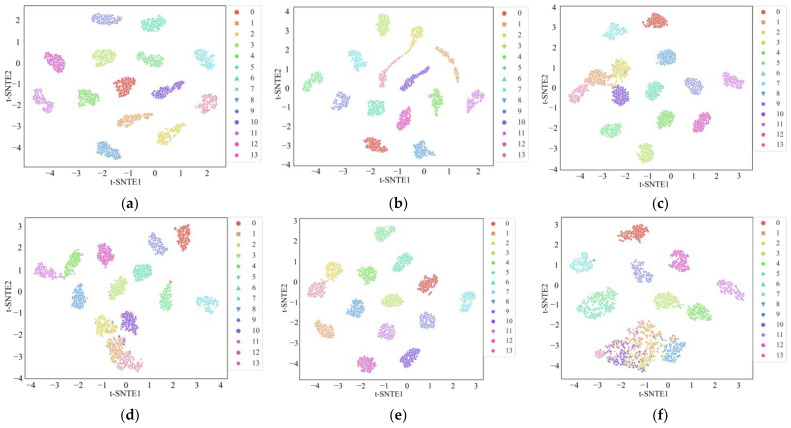
The visualization of the features of the fully connected layer using t-SNE on the PMSM stator dataset. (**a**) MWAL-CNN, (**b**) MWA-CNN, (**c**) MA1DCNN (**d**) 1DCNN, (**e**) ResNet, (**f**) MLP.

Similarly, the confusion matrix for the proposed model and the t-SNE plots for different models are provided in Figure 14 and Figure 15, respectively. In the confusion matrix, only one misclassification occurred in class 2, indicating that the model classification performance remained excellent. According to the t-SNE plots, several models were able to separate different stator faults. However, other models still exhibited some instances of misclassification. MLP, in particular, exhibited two clusters with significant overlap. In contrast, the proposed model attained the best performance, demonstrating that the fusion of three-phase currents contains richer information.

#### 4.3.3. Validity of Multi-Current Signals

**Figure 16:**
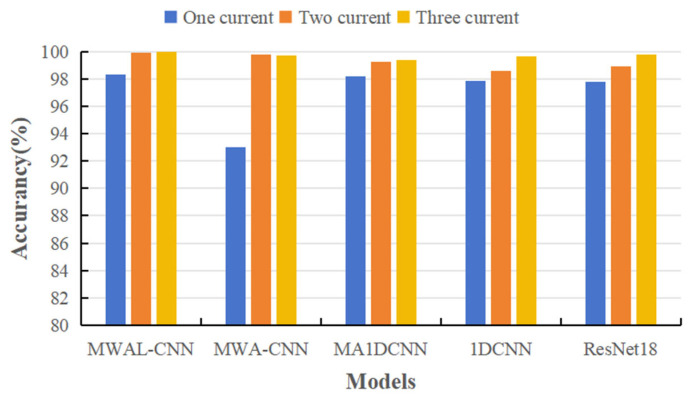
The experimental results of the five methods for different signals on the PMSM stator dataset.

The PMSM stator dataset comprised current signals from standard three-phase currents, the PU bearing dataset comprised current signals from standard three-phase currents, and the PU bearing dataset comprised two-phase currents. Comparative experiments were conducted using single-phase, two-phase, and three-phase current datasets to demonstrate the model’s ability to fuse multiple current signals, as shown in Table 7 and Figure 16. According to the results, the current signals were able to effectively classify different stator fault types on simpler datasets. The bar chart indicates that the performance of single-phase currents was significantly lower than that of two-phase and three-phase currents. However, with respect to the MWA-CNN model, the accuracy of three-phase current signals was higher than that of two-phase currents, demonstrating better performance.

#### 4.3.4. DWT Layer Validity

The effectiveness of different modules relative to the stator fault dataset is validated in this section, following the same experimental configuration as in Section 4.2.4. The results are shown in Table 8 and are generally consistent with those from the PU dataset. However, one notable difference is that, in the case of simpler data, where only the high- and low-frequency DWT modules were added, the performance was not as outstanding as in the case of the PU dataset, particularly for the three-phase current scenario. Here, the improvement from various modules was relatively small.

**Figure 17:**
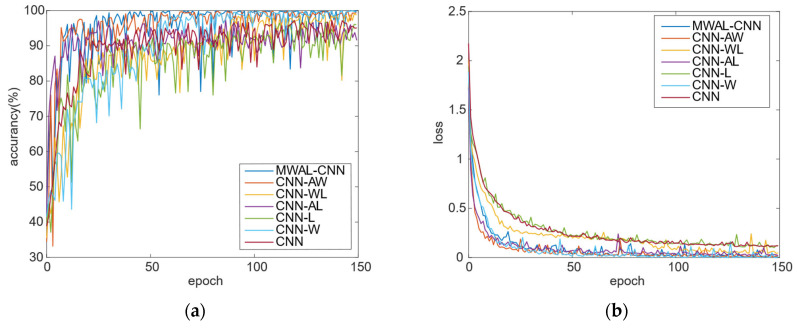
The validation accuracy curves and training loss curves of seven models obtained with the PMSM stator dataset: (**a**) validation accuracy curve; (**b**) training loss.

Furthermore, the accuracy curve for the test set and the loss curve for the training set are provided in Figure 17. The results, again, are quite similar to those obtained with the PU dataset. However, one noticeable difference is that the vibration amplitudes were smaller in this dataset, resulting in less pronounced overfitting. The curves were smoother and converged more quickly compared to those obtained with the PU dataset. The advantage of the DWT module for high- and low-frequency separation was not as prominent. Additional significant oscillations occurred when the module with the weight-updated LSTM layer was added, especially in datasets with fewer data points where even minor weight changes could lead to more significant fluctuations in the model’s accuracy. This highlights the importance of the synergy among the three modules.

### 4.4. Computational Burden Analysis

This section analyzes the computational burden of the proposed MWAL-CNN and six other network models. All of them, except for the MWAL-CNN, had five or six CNN layers, and Table 8 shows the parameters and Multiply Accumulate Operations (MAC) of these methods. From the table, it can be seen that, although the added DWT layer and hybrid attention layer of MWAL-CNN increased the model computation, owing to it containing only three layers of cyclic DWT and CNN compared to the five CNN layers found in the other models, the proposed model had low MACs of only 12.86 M, and the parameter was only 706.31 K, which is a slight reduction relative to the other models.

MWAL-CNN was able to train a single batch on the PU dataset in 10.69 s, with a total of 35,064 training samples. It also trained a single batch on the PMSM synchronous stator fault dataset in 3.16 s, with a total of 13,504 training samples, which is an acceptable time relative to the improvement in the accuracy rate. However, when MWAL-CNN was embedded in the fault diagnosis system, and when the data volume increased, the training time also increased by about 3 s for each additional 10,000 samples, and the training time doubled for each additional fault condition or faulty device. However, when the fault data included more than 90,000 samples, the training time of a single batch of MWAL-CNN was close to 30 s, a time that is too long. Therefore, federated learning could be used to distribute the data of different working conditions or other ways to improve the training efficiency.

### 4.5. Discussion

In this paper, two case studies were assessed using the benchmark motor bearing dataset and the permanent magnet synchronous motor stator fault dataset to validate the effectiveness and practicality of the proposed MAWL-CNN method for collecting two-phase and three-phase current data.

The experimental results demonstrated that the MAWL-CNN model achieved recognition rates of 99.772% and 99.959% on these two datasets, respectively, indicating that MAWL-CNN can deliver excellent diagnostic results in these scenarios. It is worth noting that MAWL-CNN significantly outperformed five other competitive methods, highlighting the superiority of the proposed approach.

Comparative experiments were conducted using single, double, and triple signal combinations to investigate the model fusion effect for different currents with different current fusion effects. The results indicate that three-phase signals yield the best performance. Moreover, two-phase currents can also better identify the location of anomalies. Ablation studies were performed to verify the effectiveness of the remaining high- and low-frequency separation DWT layer, the hybrid attention layer, and the weight-updated LSTM layer, showing that each layer is indispensable. It is expected that the proposed method could be successfully applied to industrial scenarios of permanent magnet synchronous motors with low speed and high torque, and could be embedded into fault monitoring systems for anomaly detection of permanent magnet synchronous motors, and the memory of the fault monitoring system should be more than 8 G and 4 core CPU.

To study the model computation time and model complexity, the per-epoch model running time and MAC were used as evaluation indices. The experimental results show that, with the limited number of datasets in the open dataset, the computation time added by the DWT hybrid attention mechanism of MAWL-CNN is short. The MWAL-CNN runs on both datasets in a controllable running time range, with lower model complexity.

## 5. Conclusions

In this paper, the detection of faults in permanent magnet synchronous motors was investigated using multi-current fusion. Therefore, a new method for weight-updated LSTM anomaly detection with DWT-CNN feature fusion was proposed. The method extracts rich fault features using DWT and CNN networks with low-frequency simple encoding and high-frequency large kernel convolution denoising based on the possibility of detecting two-phase or three-phase currents in an industrial setting. The proposed features are extracted by a hybrid attention mechanism in frequency and time domains to extract additional insignificant features and fused to feed into weight-updating LSTM networks for anomaly detection. The real motor bearing dataset and stator fault dataset were used to validate the proposed method. Experimental results demonstrate that the DWT-CNN can fuse multi-current signals, outperforming vibration signals, with the MWAL-CNN method exhibiting superior performance compared to other methods. Moreover, despite the remarkable achievements of multi-sensor fusion, adding extra data dimensions increases the computational burden. In future work, the embedding of MWAL-CNN into a PMSM fault monitoring system should consider PMSM type and data computation volume issues. We will further employ transfer learning for migrating diagnostics from publicly available datasets to PMSM, and federated learning for parallel training of data to reduce model complexity and increase data confidentiality.

## Figures and Tables

**Table 1:** Description of the PU dataset Information.

Arrangement Damage	Bearing Element	Extent of Damage	Label
Pitting	Single point on outer ring	1 single damage	1
Indentations	Single point on outer ring	1 single damage	2
Random pitting	Single point on outer ring	2 repetitive damage	3
Pitting	Single point on outer ring	2 single damage	4
Random indentations	Distributed on outer ring	1 repetitive damage	5
Random pitting	Single point on (outer ring) and inner ring	2 multiple damage	6
Pitting	Distributed on (outer ring) and inner ring	3 multiple damage	7
Random indentations	Distributed on outer ring and inner ring	1 multiple damage	8
Pitting	Single point on inner ring	1 multiple damage	9
Pitting	Single point on inner ring	1 multiple damage	10
Pitting	Single point on inner ring	3 single damage	11
Random pitting	Single point on inner ring	1 repetitive damage	12
Pitting	Single point on inner ring	2 single damage	13
Pitting	Single point on inner ring	1 single damage	14

**Table 2:** The experimental results of MWAL-CNN and five comparison methods on the PU dataset.

Method	Accuracy (%)	Precision (%)	Recall (%)	F1 Score (%)	Times (s)
MWAL-CNN	99.772	99.792	99.786	99.786	10.69
MWA-CNN	98.774	98.808	98.774	98.770	9.37
MA1DCNN	95.696	95.994	95.692	95.599	9.07
1DCNN	94.527	95.195	94.515	94.521	3.84
ResNet18	98.575	98.756	98.571	98.576	10.775
MLP	85.120	85.643	85.092	85.143	2.94

**Table 3:** The experimental results of MWAL-CNN and four comparison methods for different signals on the PU dataset.

Method	Accuracy (%)			F1 Score (%)		
	Vibration	One Current	Two Current	Vibration	One Current	Two Current
MWAL-CNN	98.774	99.601	99.772	98.775	99.601	99.786
MWA-CNN	97.577	92.189	98.774	97.566	92.032	98.770
MA1DCNN	98.404	79.532	95.696	98.410	79.200	95.599
1DCNN	93.415	87.200	94.527	93.475	86.576	94.521
ResNet18	98.176	95.952	98.575	98.157	95.941	98.576

**Table 4:** The ablation experimental results of MWAL-CNN.

	CNN	CNN-W	CNN-L	CNN-AL	CNN-WL	CNN-AW	MWAL-CNN
Average accuracy (%)	63.797	97.434	80.017	74.059	97.948	98.290	99.772
F1 score (%)	60.656	97.439	80.212	72.223	97.940	98.282	99.86

**Table 5:** Description of the PMSM stator dataset information.

Defect	Dimension	Motor Power	Speed	Label
No defect	0	1000 W	100 KHz	1
Inter-turn short circuit fault	3.35% severity	1000 W	100 KHz	2
Inter-turn short circuit fault	8.74% severity	1500 W	100 KHz	3
Inter-turn short circuit fault	9.81% severity	3000 W	100 KHz	4
Inter-turn short circuit fault	16.08% severity	1500 W	100 KHz	5
Inter-turn short circuit fault	21.69% severity	1000 W	100 KHz	6
Inter-coil short circuit fault	3.93% severity	1000 W	100 KHz	8
Inter-coil short circuit fault	7.02% severity	1500 W	100 KHz	9
Inter-coil short circuit fault	7.12% severity	3000 W	100 KHz	10
Inter-coil short circuit fault	23.20% severity	1500 W	100 KHz	11
Inter-coil short circuit fault	23.48% severity	3000 W	100 KHz	12

**Table 6:** The experimental results of MWAL-CNN and five comparison methods on the PMSM stator dataset.

Method	Accuracy (%)	Precision (%)	Recall (%)	F1 Score (%)	Times (s)
MWAL-CNN	99.959	99.959	99.959	99.959	3.16
MWA-CNN	99.715	99.718	99.715	99.715	2.76
MA1DCNN	99.389	99.393	99.389	99.389	2.53
1DCNN	99.674	99.678	99.674	99.673	1
ResNet18	99.796	99.801	99.796	99.800	2.47
MLP	89.984	89.309	88.970	88.684	0.81

**Table 7:** The experimental results of MWAL-CNN and four comparison methods for different signals on the PMSM stator dataset.

Method	Accuracy (%)			F1 Score (%)		
	One Current	Two Current	Three Current	One Current	Two Current	Three Current
MWAL-CNN	98.290	99.924	99.959	98.286	99.923	99.959
MWA-CNN	92.997	99.783	99.715	93.002	99.778	99.715
MA1DCNN	98.168	99.267	99.389	98.173	99.265	99.389
1DCNN	97.883	98.575.	99.674	97.874	98.571	99.673
ResNet18	97.801	98.941	99.796	97.797	98.930.	99.800

**Table 8:** The parameters and computational burden of MWAL-CNN and the five comparison methods.

	MWAL-CNN	MWA-CNN	MA1DCNN	1DCNN	ResNet18	MLP
Parameters	706.31 K	1.39 M	326.97 K	182.25 K	3.85 M	11.72 M
MACs	12.86 M	59.85 M	76.64 M	18.71 M	175.56 M	2.8 M

## Data Availability

Data are contained within the article.

## Appendix A

**Table A1:** Detailed explanatory table of professional terms.

Abbreviations	Full Name of the Term
PMSM	Permanent Magnet Synchronous Motor
DW-CNN	Discrete Wavelet Transform Convolutional Neural Networks
DWT	Discrete Wavelet Transform
MWAL-CNN	Multilayer Wavelet Attention LSTM Convolutional Neural Network
FFT	Fast Fourier Transform
STFT	Short Time Fourier Transform
CWT	Continuous Wavelet Transformation
scSE	Spatial-Channel Sequeeze and Excitation
cSE	Spatial Squeeze and Channel Excitation
sSE	Channel Squeeze and Spatial Excitation
FAM	Channel Frequency Attention Mechanism
MWA-CNN	Multilayer Wavelet Attention Convolutional Neural Network
MA1DCNN	Multiattention One-Dimensional Convolutional Neural Network
1DCNN	One-Dimensional Convolutional Neural Network
ResNet	Deep residual network
Db wavelet	Daubechies Wavelet
t-SNE	t-distributed Stochastic Neighbor Embedding
MAC	Multiply Accumulate Operations

**Table A2:** Detailed explanatory table of professional symbol.

Symbol	Explanation
*x*[*n*]	The real sampled signal
*g*[*n*]	High-Frequency Component (High Scale)
*h*[*n*]	Low-Frequency Component (Low Scale)
[C,H,W]	Input data dimensions
U	Feature
σ	Sigmoid
*h*(*t*)	Output at time t
*c*(*t*)	Cell state at time t
tanh	Tanh Neural Network Layer
*x*(*t*)	Input at time t
Conv1d	One-dimensional convolutional neural network with different kernels
Scon1d	Standard one-dimensional convolutional neural network
Attention	Hybrid Attention Mechanisms
BN	Batch Normalization
Groupnorm	Group Normbalization
Relu	Rectified Linear Unit
cat	Torch.cat()
